# Hospital and Institutional News

**Published:** 1918-08-17

**Authors:** 


### HOSPITAL AND INSTITUTIONAL NEWS.

### HER MAJESTY'S VISIT TO BRIGHTON.

Her Majesty the Queen, accompanied by their Royal Highnesses Prince George and Princess Mary, paid a visit on Friday, August 9, to the Queen Mary's Workshops and the Pavilion General Hospital, Brighton. They arrived by motor-car, and were met at the entrance to the Pavilion by the Commandant, Colonel G. T. K. Maurice, C.M.G., A.M.S. After the matron and the medical staff had been presented the Royal party went through the building to the workshops. Entering by the shoemakers' shop Her Majesty passed in succession through the carpenters' shop, the electrical shop, the engineering shop, the clerical shop, the tailors' shop, and the mechanical drawing department. Her Majesty showed the greatest interest in the work going on, and conversed with many of the pupils learning trades. The learners were highly delighted by her gracious manner and the high honour done to them. After a thorough inspection of the workshops, the Queen signified her wish to visit the wards. She was conducted by the Commandant through A section, and thence to the plaister-room, the department in which temporary artificial legs are made, as described in recent numbers of The Hospital. Lady Shifner, the head of this department, was presented by the Commandant, and thereafter showed Her Majesty the details of this most excellent and useful work. Her Majesty and the Royal party were greatly interested. In response to Her Majesty's desire Lady Shifner moulded the first stage of a plaister leg on the stump of one of the patients who were waiting treatment in the plaister-room. Her Majesty also inspected some of the false legs made in the department, and expressed her satisfaction at the clever work done. A visit was then paid to the Dome. On the way Her Majesty passed by the serried ranks of patients and spoke to several, evincing a special interest in one patient, a man who sports the ribbons of the Military Medal and the Mons Star, hard-earned distinctions, for he was five times wounded before a shell deprived him of both his legs. After passing through the Dome and the riding-school, and speaking to many patients who were in their beds there and in the garden outside, Her Majesty passed through the grounds to the waiting cars and departed, after expressing her gracious approval of all she had seen.

### A TOUR OF BELFAST HOSPITALS.

Lord French, on his recent visit to Belfast, spent most of his time in a tour of the Belfast hospitals. The Ulster Volunteer Force Hospital was the first visited, where, in company with the chairman, Lieutenant-General Sir George Richardson, he inspected the curative workshops where the limbs for orthopædic cases are made and fitted. The next hospitals to be visited were the Royal Victoria and the Hilden Convalescent institutions. In the latter the Lord-Lieutenant talked with several men who had been through the retreat from Mons. The last institution to be inspected was the Craigavon Neurasthenic Hospital, a branch of the Ulster Volunteer Force Hospital, which had the honour of being the first hospital to be visited earlier in the day.

### HIGHBURY AS A PERMANENT HOSPITAL.

Mr. Austen Chamberlain has presented Highbury, his father's famous house at Moor Green, to the City of Birmingham. The land attached is to be sold at the Government valuation, and the house itself, which has been used for three years as a V.A.D. military hospital, will become a permanent hospital for chronic orthopædic cases. The house was built in 1880 from the plans of the statesman's friend, Mr. J. H. Chamberlain, and Mr. Milner, the landscape gardener, laid out the grounds. Within, the library, with its panelling and oak ceiling, is the most remarkable feature, and without are the glass-houses where the famous orchids were grown.

### A LEICESTER LADY'S GENEROUS BEQUESTS.

Many generous bequests to Church and charitable institutions are contained in the will of the late Miss Ada K. A. Bickersteth, niece of Canon Vaughan, formerly Vicar of St. Martin's, Leicester. The Vaughan Working Men's College is to receive £5,000, until such time as the college is taken over by the Local Education Committee, when the bequest falls into residue. Other local bequests of interest are £5,000 to the Peterborough Diocesan Trustees, the income to be paid to the Vicar of St. Martin's in augmentation of the income of the living, £1,000 to the Leicester Archidiaconal Church Extension Fund, £500 to the Royal Infirmary, £500 to the Stoneygate Home, and £100 to the District Nursing Association. Other bequests are £5,000 to the Queen Victoria Clergy Fund, £5,000 to the London-Over-the-Border Church Fund, £1,000 each to the Clergy Pensions Institute, the St. Albans Diocesan Poor Benefices Fund and to the Homes of St. Barnabas, £500 each to the Watford District Hospital, the Railway Benevolent Society, the Meath Home for Epileptics, the Indian Church Aid Association, the Universities Mission to Central Africa, and to the Middlesex Hospital for the Cancer Wards, and £100 to the Sharkey Memorial School, Mussulipatat, India. After bequests to relatives, servants, and other friends, the residuary estate is bequeathed to the Melanesian Mission. The gross value of the estate is upwards of £92,000, and the residuary bequest is probably of the value of between £50,000 and £60,000.

### A CANON IN THE RING.

We do not recall any hospital besides the Alexandra at Woodhall Spa which can claim a canon for its secretary, nor are there many canons who would attend a famous boxing match at the National Sporting Club to address the remarkable audience which is to be found there on a hospital's behalf. This, however, was accomplished with great success by the Rev. Canon J. O. Stephens, hon. secretary of the Alexandra Hospital, Woodhall Spa, when £126 was raised by his five minutes' address from the ring. When we venture to add that Canon Stephens has passed his eighty-fifth year, it will be agreed that he is an example of resource to many younger hospital secretaries.

### SECTIONAL SUBSCRIPTIONS AT EDINBURGH.

A new form of collecting sectional subscriptions has been started in Edinburgh by the organisation of a "League of Subscribers to the Edinburgh Royal Infirmary." The managers and the Edinburgh Trades Council have agreed by this means to organise subscriptions from employees in the public works and establishments of the city and district. Membership will be voluntary, and will consist in an agreement to subscribe at least a penny a week from an adult and a halfpenny a week from each apprentice for one year from the time of enrolment. It is stated that membership releases a person from any other obligation to subscribe. It is open to renewal every year. If the plan succeeds, there will be 40,000 adults and 20,000 apprentices whose pennies and halfpennies will amount to £10,000 a year. If we mistake not, the essence of the plan is to apply the principles of the workmen's funds to those who, not being engaged in factories, are employed in public works and the retail trade of the city and its neighbourhood.

### WOMEN MEDICAL STUDENTS AT UNIVERSITY COLLEGE HOSPITAL.

We are officially informed that the General Committee of University College Hospital have taken the important step of deciding to admit women as medical students to the hospital and medical school on and after October 1, 1918. Women have been admitted to the Faculty of Sciences at University College since 1877, and to the Faculty of Medical Sciences since October 1917. They will now be able to pursue the whole of their medical education at University College and University College Hospital, and will be in a position to avail themselves of the new arrangements which have been entered upon with the National Hospital for Nervous Diseases, Queen Square; the Children's Hospital, Great Ormond Street; and the Central London Ophthalmic Hospital, Judd Street, which when completed will enable the students of University College Hospital to carry out a portion of their clinical studies at these special hospitals.

### THE TRENCH-FEVER PROBLEM.

Speaking recently before the Royal Society of Medicine, Colonel William Hunter described trench fever as one of the most important problems in preventive medicine, and said that quite 10 per cent. of the medical cases taken into hospital were due to it. All to whom the facts are familiar are in agreement with Colonel Hunter's view of the importance of the matter, but the percentage of cases mentioned in the reports of his address was not generally properly appreciated and understood. A little further inquiry into the question elicits the information that about 40 per cent. of trench sickness is attributable to trench fever, and of these cases approximately 50 per cent. are treated at the front and are thence returned to duty. The other 50 per cent. go to the base, with the result that, roughly, a similar division there takes place. About half these cases are returned to duty from the base. The remaining half are transferred to England, and the latter 50 per cent., which would represent 10 per cent. of trench sickness, is probably the subject of Colonel Hunter's reference.

### A MEDICAL OFFICER ON EDUCATION.

In connection with the Welsh National Eisteddfod a conference was arranged, at which the principal feature was an outspoken address by the medical officer of health for Swansea, Dr. Thomas Evans. Dr. Evans appealed for a new conception of the training of medical men in preventive medicine, which should deal rather with the maintenance of health than with the cure of disease. He urged the authorities of the new National Medical School at Cardiff to bring this about. He made a strong appeal for the opening in the colleges of a new department of physical education, where the first year of the teacher's life would be concerned with physiology, hygiene, gymnastics, dancing, and games. The first part of the address is likely to be more acceptable than the latter, for the unfortunate schoolmaster is inclined to groan beneath the burden of subjects which he is nowadays invited to study and to teach.

### CHINESE MEDICAL WOMEN.

The war has provided many openings in this country for foreign medical men and women. The latest such appointment is that of Miss Martha Hunter Hoa Hing, a Chinese medical woman, who has been chosen house surgeon at the Alexandra Hospital for Children, Brighton. Miss Hoa Hing has been in England for some seven years, and has a brother in the British Army. She took her diplomas here—L.R.C.P. and L.R.C.S. Edinburgh, and L.R.F.P.S. Glasgow—in 1916. The circumstances in which institutions now find themselves when a vacancy on the medical staff arises is typified by the case of the Shoreditch Board of Guardians. In answer to their recent advertisement for an assistant medical officer, all the applications received, apart from one from a Chinese, were from coloured medical men.

### A SEVENTY-SEVENTH ANNUAL MEETING.

The seventy-seventh annual meeting of the Medico-Psychological Association of Great Britain and Ireland was held at Edinburgh on July 23 and 24, 1918, under the presidency of Lieut.-Col. John

Keay, R.A.M.C. After the transaction of the usual business the President delivered his inaugural address. This was followed by papers on "The Prevention and Treatment of Neurasthenia and other Functional Nervous Breakdowns," by Dr. Claud F. Fothergill, and "The Infective Factors in Some Types of Neurasthenia," by Dr. W. Ford Robertson. On the second day the members and their friends were hospitably entertained by Lieut.-Col. and Mrs. Keay to luncheon and an "At Home" at the Edinburgh War Hospital, Bangour. The wards of this excellently equipped hospital were visited and demonstrations were given by Lieut.-Col. Cathcart, R.A.M.C. (T.), and Major Rankine on the construction and fitting of provisional artificial limbs; by Lieut.-Col. Sir Harold J. Stiles, R.A.M.C., on interesting cases from the orthopædic department; by Capt. Edwin Bramwell, R.A.M.C., on cases illustrating functional neuroses. Major D. G. Marshall, I.M.S., and Dr. L. K. Davies showed microscopic preparations illustrating malaria and dysentery. By the kindness of the chairman and managers and Dr. G. M. Robertson an "At Home" was held at the Royal Edinburgh Mental Hospital, Morningside. Owing to the war the annual dinner was not held, but the well-known hospitality of the Edinburgh members contributed in no small measure to the success of a very enjoyable and instructive meeting.

### ACCIDENT CASES AT POOR-LAW INFIRMARIES.

Dr. W. L. MacCormac, medical superintendent of St. James's Infirmary, Wandsworth, shows in his annual report the gradual development of the modern Poor-Law infirmary into a general hospital. The admissions during the year numbered 4,271, these including 370 military patients; 329 accident cases were admitted, or, on an average, nearly one a day, whilst 1,115 operations were performed. The deaths were 899, and of these 108 were babies under one year old and 236 people over seventy. If the latter figures are a testimony to the longevity which has been characteristic of life in the healthy borough of Wandsworth, with its many acres of open commons, the former show the useful and varied work performed by a modern infirmary.

### A WARD IN A WORKSHOP.

A new ward of the West End Hospital, Welbeck Street, has been opened by Admiral Sir Rosslyn Wemyss for Navy and Army pensioners suffering from shell shock or nervous disease. The ward consists of two workshops on the premises of Debenham and Freebody, whose shop adjoins the hospital. After conversion the new ward is able to accommodate twenty-two patients, which brings the total number in the hospital to 122. Sir Rosslyn, in opening the ward, which has been named after the donor, said that he had come to show his sympathy with the work of the medical staff and the nurses, for the senior officers in the Services realised the need for an increase of hospital accommodation.

### A CONVALESCENT HOSPITAL CLOSED.

After three and a half years the convalescent hospital at North Hall, East Chiltington, Sussex, has been obliged to close down, through the ill-health of the owner, the Hon. Mrs. Bampfylde, the difficulty of securing coal, the rationing restrictions, and the general rise in the cost of provisions. Situated in the country depths of the Sussex Weald, a few miles from Lewes, the hospital has done good work for the 800 patients who have passed through it; and they with the quartermaster, Mrs. Gwynneth Roberts, the staff of eighteen V.A.D. nurses, and Mrs. Rudolf Besier, the masseuse, will leave it with regret. Dr. Fawsett, of Lewes, has acted as honorary medical officer from the first, a voluntary work which has rarely meant less than a daily visit.

### NAMING A BED AFTER A SECRETARY.

Mr. Addison Greene, who has held for eight years the post of honorary secretary to the Hornsey Cottage Hospital, has resigned the position in consequence of the distance of his home from the hospital. It is remarkable that Mr. Greene was an active worker for the hospital from the time when it was planned and before it was actually built. In acknowledgment of his services a bed has been named after him. This is something of a precedent, for the officers of a hospital are rarely honoured by a memorial, which is usually reserved for those who make a substantial gift. Where services have been exceptional we think the honour fit and timely, for hospital tradition is fostered by the perpetual association of the personality of those who have worked within its walls, which should record the names as they are echoed to the feet of these enthusiasts. Mr. George Pain, who has been acting as assistant, has now accepted the post of honorary secretary.

### SHOULD RED CROSS HOSPITALS BE RATIONED?

Lady Cavendish Bentinck complains to the *Morning Post* that the Joint War Committee appointed by the British Red Cross Society and the Order of St. John of Jerusalem are more severe than the Food Controller himself in the rationing of the auxiliary hospitals under their care. When the general public are rationed in respect of six articles only, it is held to be vexatious that the Red Cross hospitals have to keep within certain limits in supplies of no fewer than eighteen. Why should the general public have unlimited supplies when men who have fought and bled for their country are tied down to so many ounces per day? Two reflections present themselves which lead one to suspect that there are fallacies in Lady Cavendish Bentinck's arguments. It is true that the public are rationed in the matter of few articles of food, but the system of registration in most districts forbids anything like an unlimited supply of other foods, especially such things as cheese, tea, milk, and bacon. Moreover, hospitals—at least properly managed hospitals—have always been rationed in peace-time as well as in war-time, and the diet scale for staff as well as patients is an instrument which has been used for many years. The only genuine complaint is one in respect of a niggardly diet-scale, and this would soon reveal itself through the progress of the patients and the health of the staff.

### THIS WEEK'S DRUG MARKET.

There has been a little more life in the market this week, with a good undercurrent of inquiry. There is no change in the position of potassium bromide. A large demand for Epsom salts, Glauber salts, and heavy carbonate of magnesia continues, and English makers cannot cope with half the orders received. English refined camphor is scarce and a further advance in price is probable; the demand for tablets is especially good. Cloves are again dearer, and probably the oil will follow suit. Barbitone is offered at slightly lower rates. Imported saccharin has a rising tendency in price owing to the continued large demand. Oil of caraway seed is dearer. Fair business has been done in eucalyptus oil. There is a slight improvement in the demand for tartaric and citric acids and the price tendency is slightly upwards. An upward movement in the price of salicylic acid is not improbable in view of the advance in the price of carbolic acid; supplies of salicylic acid, however, are good, and it is not anticipated that the advance, if any, will be very substantial. The value of quinine is well maintained.

